# Predictors of PrEP awareness, PrEP discussion and interest in long‐acting injectable PrEP among Filipina transfeminine adults

**DOI:** 10.1002/jia2.26080

**Published:** 2023-06-12

**Authors:** Arjee Javellana Restar, Ma Irene Quilantang, Jeffrey Wickersham, Alex Adia, John Guigayoma, Amiel Nazer Bermudez, Omar Galárraga, Dalmacio Dennis Flores, Susan Cu‐Uvin, Jennifer Nazareno, Don Operario, Olivia Sison

**Affiliations:** ^1^ Department of Epidemiology University of Washington School of Public Health Seattle Washington USA; ^2^ Department of Behavioral and Social Sciences, Research Education Institute for Diverse Scholars (REIDS) Yale University School of Public Health New Haven Connecticut USA; ^3^ Philippines Health Initiative for Research, Service & Training (PHIRST) Brown University School of Public Health Providence Rhode Island USA; ^4^ Department of Behavioral and Social Sciences Brown University School of Public Health Providence Rhode Island USA; ^5^ Department of Behavioral Sciences University of Philippines‐Manila Manila Philippines; ^6^ Department of Internal Medicine Yale University School of Medicine New Haven Connecticut USA; ^7^ Division of Health Policy and Management University of California – Berkeley School of Public Health Berkeley California USA; ^8^ Department of Epidemiology Brown University School of Public Health Providence Rhode Island USA; ^9^ Department of Epidemiology and Biostatistics University of Philippines‐Manila Manila Philippines; ^10^ Department of Health Services, Policy, and Practice Brown University School of Public Health Providence Rhode Island USA; ^11^ Department of Family and Community Health University of Pennsylvania School of Nursing Philadelphia Pennsylvania USA; ^12^ Department of Behavioral, Social, and Health Education Sciences Emory University Rollins School of Public Health Atlanta Georgia USA; ^13^ Institute of Clinical Epidemiology, National Institutes of Health University of Philippines‐Manila Manila Philippines

**Keywords:** HIV, PrEP, transfeminine, transgender, Philippines, implementation

## Abstract

**Introduction:**

Transfeminine adults are impacted by the HIV epidemic in the Philippines, and newly approved modalities of pre‐exposure prophylaxis (PrEP), including long‐acting injectable (LAI‐PrEP), could be beneficial for this group. To inform implementation, we analysed PrEP awareness, discussion and interest in taking LAI‐PrEP among Filipina transfeminine adults.

**Methods:**

We utilized secondary data from the #ParaSaAtin survey that sampled Filipina transfeminine adults (*n* = 139) and conducted a series of multivariable logistic regressions with lasso selection to explore factors independently associated with PrEP outcomes, including awareness, discussion with trans friends and interest in LAI‐PrEP.

**Results:**

Overall, 53% of Filipina transfeminine respondents were aware of PrEP, 39% had discussed PrEP with their trans friends and 73% were interested in LAI‐PrEP. PrEP awareness was associated with being non‐Catholic (*p* = 0.017), having previously been HIV tested (*p* = 0.023), discussing HIV services with a provider (*p*<0.001) and having high HIV knowledge (*p* = 0.021). Discussing PrEP with friends was associated with older age (*p* = 0.040), having experienced healthcare discrimination due to transgender identity (*p* = 0.044), having HIV tested (*p* = 0.001) and having discussed HIV services with a provider (*p* < 0.001). Very interested in LAI‐PrEP was associated with living in Central Visayas (*p* = 0.045), having discussed HIV services with a provider (*p* = 0.001) and having discussed HIV services with a sexual partner (*p* = 0.008).

**Conclusions:**

Implementing LAI‐PrEP in the Philippines requires addressing systemic improvements across personal, interpersonal, social and structural levels in healthcare access, including efforts to create healthcare settings and environments with providers who are trained and competent in transgender health and can address the social and structural drivers of trans health inequities, including HIV and barriers to LAI‐PrEP.

## 1 INTRODUCTION

Transfeminine adults are disproportionately impacted by the growing HIV epidemic in the Philippines [1]. Based on successful findings from an in‐country pre‐exposure prophylaxis (PrEP) demonstration study showing clinical effectiveness [2], the Philippines’ Department of Health recently approved tenofovir/emtricitabine as daily oral PrEP for HIV prevention [3]. Updated national clinical guidelines now recognize PrEP as part of its national essential medicine formulary as of January 2022 [3], and recommend the urgent need to deliver it to populations placed vulnerably to HIV infection, including Filipina transfeminine adults who are bearing the brunt of the epidemic—with HIV prevalence approximately doubling (3.9% in 2020 vs. 1.7% in 2018) within a short timeline [1, 4]. Moreover, with several new PrEP modalities in the clinical trial pipeline, like long‐acting injectable pre‐exposure prophylaxes (LAI‐PrEP) [5, 6], understanding how LAI‐PrEP would fit into the lives of this population is necessary for the future in‐country PrEP implementation programming.

Determinants of PrEP implementation are multilevel [7], and frequent barriers included factors (1) at the personal level—lack of PrEP awareness and knowledge [8, 9, 10, 11]; (2) at the interpersonal level—discomfort talking to providers about PrEP, provider's lack of knowledge or support of PrEP and having mistrust of providers [12, 13, 14]; (3) at the social level—lack of communication about PrEP among community members [14, 15, 16], including PrEP discussion among trans friends and providers [14]; and (4) at the structural level—having segregated or fragmented healthcare systems, concern about paying for PrEP, including out‐of‐pocket costs, stigma and discriminatory practices and policies related to PrEP, HIV, transgender identity and sexual behaviour, as well structural determinants, such as poverty, unemployment, unstable housing [13, 15, 16, 17, 18].

To our team's knowledge, there are currently no formative studies that specifically examine PrEP implementation indicators among Filipina transfeminine populations [19]. Given this need, the purpose of our exploratory study is to examine prevalence as well as social barriers and opportunities related to outcomes along the PrEP continua, particularly awareness, discussion among friends and interest in taking LAI‐PrEP.

## 2 METHODS

### 2.1 Study sample and procedures

We utilized the STROBE checklist for reporting cross‐sectional study (Supporting Information File, Table S1). This is a secondary data analysis of Filipina transfeminine respondents (*n* = 139) from the #ParaSaAtin project, a cross‐sectional self‐reported online survey conducted between June 2018 and May 2019. Full study procedures are described elsewhere [20]. Briefly, the study used online convenience sampling recruitment strategies, which included social media advertisements, and online social groups (e.g. Facebook, Twitter) hosted by local HIV community‐based organizations and transgender communities in the Philippines. Participants enrolled in the study self‐reported to be: 18 years or older, transfeminine (i.e. have a gender identity along the transfeminine spectrum, such as women, trans women and with sex assigned at birth as male), had condomless sex within the past year, currently living in Metro Manila and Central Visayas, and demonstrated English and consent comprehension via a brief cognitive screening form. The survey lasted between 20 and 25 minutes, and participants received ₱300 ($5.85 USD) for completing the survey. Electronic, written consent forms were obtained from all participants in this study.

### 2.2 Measures

#### 2.2.1 Demographic factors

All participants reported their gender identity as woman (woman, trans woman, transfeminine and have sex assigned as birth as male). We asked participants about their age, current living location, highest education attained, past year income, religious affiliation and sexual orientation.

#### 2.2.2 Social and community‐level factors

We assessed whether participants had a history of homelessness (yes vs. no), were currently unemployed (yes vs. no) or recently (within the past 4 months) had engaged in sex work themselves (yes vs. no). We adapted Lippman and colleagues’ social cohesion scale [21], which is comprised of 9 items with 5‐point Likert responses (from strongly agree = 1 to strongly disagree = 5). Summed scores (Cronbach α= 0.87) were dichotomized into low versus high social cohesion. We then assessed social participation in general and lesbian, gay bisexual, and transgender (LGBT) community activities by adapting Fonner and colleagues’ 4‐item social participation scale [22]. Similarly, summed scores (Cronbach α= 0.75 for general social participation and Cronbach α= 0.76 for LGBT social participation, respectively), were dichotomized into low versus high social participation. Lastly, we adapted Haggerty and colleagues’ 6‐item healthcare accessibility scale [23]. Summed scores (Cronbach α= 0.93) were also dichotomized into poor/fair versus good/excellent accessibility.

#### 2.2.3 HIV and other healthcare indicators

We asked if participants have current health insurance, ever took hormones for gender affirmation, ever had surgery for gender affirmation or experienced healthcare discrimination due to their transgender identity. We also asked about their healthcare history with HIV services, including if they ever had an HIV test, discussed HIV with their provider or sexual partner, or avoided HIV services due to: cost, distance to/from a healthcare facility, their transgender identity or healthcare facility's lack of LGBT anti‐discrimination policy. All responses were either yes or no. We also used the International AIDS Questionnaire to assessed participants’ HIV knowledge (Cronbach α= 0.84), and scored responses were dichotomized into low versus high HIV knowledge categories [24].

#### 2.2.4 PrEP awareness, PrEP discussion among trans friends and interest in LAI‐PrEP (outcomes)

We adapted our PrEP questions from Restar and colleagues [12]. Participants were first provided a brief definition of PrEP: “One way to fight HIV is called PrEP, which stands for pre‐exposure prophylaxis. PrEP works by giving HIV‐negative people HIV drugs to keep them from getting HIV.” We then asked participants if they “have heard of HIV‐negative people taking HIV medication before sex because they thought it would lower their chances of getting HIV (also known as PrEP)?” We then asked if they had PrEP discussions among their trans friends (yes vs. no). Lastly, we assessed interest in LAI‐PrEP by asking, “If the possibility of having a long‐lasting injectable drug to prevent HIV (‘injectable PrEP’) was available, would you be interested in taking it?” and 3‐item Likert scale responses were recoded to aid interpretability of our logistic models, and were dichotomized to very interested versus somewhat/not at all interested.

### 2.3 Data analysis

We conducted descriptive (frequencies, percentages) and bivariate analyses to examine the distribution and independent relationships, respectively, between our exposures and outcomes: PrEP awareness, PrEP discussion and interest in LAI‐PrEP.

To examine characteristics associated with our outcomes (PrEP awareness, PrEP discussion and interest in LAI‐PrEP), we performed a series of multivariable logistic regression analyses. Given the exploratory nature of this study, we utilized lasso regression procedures to identify the key variables per outcome [25]. All variables that were selected via the lasso procedure were included in the final adjusted model per outcome. Additionally, given the modest sample size, we used non‐parametric bootstrapping procedures using 100 iterations to reduce Type 1 error per model and estimate confidence intervals [26]. Alpha for analyses was set to <0.05 *a priori*, and all analyses were conducted using StataMP version 17.0.

## 3 RESULTS

### 3.1 Sample characteristics

In our sample characteristics (Table 1), the proportion of our sample demographics, social and community‐level factors, HIV and healthcare indicators by our main outcomes are shown.

**Table 1:** Study sample characteristics of Filipina transfeminine adults (*n* = 139)

	Total	PrEP awareness		PrEP discussion among trans friends		Interest in long‐acting injectable PrEP	
	*n* (%)	Yes *n* (%)	χ2 test, *p*‐values	Yes *n* (%)	χ2 test, *p*‐values	Very interested *n* (%)	χ2 test, *p*‐values
Total	139	74 (53.24)		54 (38.85)		101 (72.66)	
Demographics							
Age							
18–24	45 (32.37)	19 (25.68)	^^^0.108	9 (16.67)	^^^0.014*	35 (36.46)	^^^0.255
25–29	54 (38.85)	29 (39.19)		27 (50.00)		37 (38.54)	
30–34	20 (14.39)	15 (20.27)		9 (16.67)		16 (16.67)	
35 years or more	20 (14.39)	11 (14.86)		9 (16.67)		8 (8.33)	
Current living location							
Metropolitan Manila	110 (79.14)	56 (75.68)	^^^0.304	40 (74.07)	0.242	75 (74.26)	^^^0.020*
Central Visayas	29 (20.86)	18 (24.32)		14 (25.93)		26 (25.74)	
Highest educational attainment							
High school or below	55 (39.57)	22 (29.73)	^^^0.041*	20 (37.04)	^^^0.920	36 (35.64)	^^^0.319
Some college	23 (16.55)	15 (20.27)		9 (16.67)		18 (17.82)	
College or beyond	61 (43.88)	37 (50.00)		25 (46.30)		47 (46.53)	
Past year income (Philippine Pesos)							
No income/less than ₱10,000	77 (55.40)	31 (41.89)	^^^0.006**	27 (50.00)	^^^0.346	50 (49.50)	^^^0.044*
₱10,000–less than P20,000	27 (19.42)	17 (22.97)		11 (20.37)		24 (23.76)	
₱20,000–less than P30,000	11 (7.91)	8 (10.81)		7 (12.96)		7 (6.93)	
₱30,000+	24 (17.27)	18 (24.32)		9 (16.67)		20 (19.80)	
Religious affiliation							
Catholic	110 (79.14)	50 (67.57)	^^^<0.001***	41 (75.93)	^^^0.404	79 (78.22)	^^^0.545
Non‐Catholic (e.g. Protestant, Christian)	21 (15.11)	19 (25.68)		8 (14.81)		17 (16.83)	
Non‐religious	8 (5.76)	5 (6.76)		5 (9.26)		5 (4.95)	
Sexual orientation							
Gay/lesbian	77 (55.40)	41 (55.41)	^^^0.866	34 (62.96)	^^^0.270	54 (53.47)	^^^0.891
Bisexual	18 (12.95)	10 (13.51)		5 (9.26)		14 (13.86)	
Straight	26 (18.71)	15 (20.27)		11 (20.37)		20 (19.80)	
Another sexual orientation	18 (12.95)	8 (10.81)		4 (7.41)		13 (12.87)	
Social and community‐level factors							
Ever homelessness							
No	101 (73.19)	49 (67.12)	^^^0.123	36 (67.92)	0.324	71 (71.00)	^^^0.396
Yes	37 (26.81)	24 (32.88)		17 (32.08)		29 (29.00)	
Currently unemployed							
No	59 (42.45)	53 (71.62)	^^^<0.001**	33 (61.11)	0.598	39 (38.61)	^^^0.178
Yes	80 (57.55)	21 (28.38)		21 (38.89)		62 (61.39)	
Recent (<4 months) sex work engagement							
No	67 (48.20)	41 (55.41)	^^^0.089	23 (42.59)	0.303	46 (45.54)	^^^0.344
Yes	72 (51.80)	33 (44.59)		31 (57.41)		55 (54.46)	
Social cohesion							
Low	66 (47.48)	23 (31.08)	^^^0.010**	16 (29.63)	^^^0.023*	37 (36.63)	0.047
High	73 (52.52)	51 (68.92)		38 (70.37)		64 (63.37)	
General social participation							
Low	86 (61.87)	39 (52.70)	^^^0.023*	29 (53.70)	0.114	62 (61.39)	0.848
High	53 (38.13)	35 (47.30)		25 (46.30)		39 (38.61)	
LGBT‐specific social participation							
Low	60 (43.17)	20 (27.03)	^^^<0.001***	16 (29.63)	0.010*	36 (35.64)	0.004**
High	79 (56.83)	54 (72.97)		38 (70.37)		65 (64.36)	
Healthcare accessibility							
Poor/fair	74 (53.24)	37 (50.00)	^^^0.025*	26 (48.15)	0.038*	55 (54.46)	0.085
Good/excellent	65 (46.76)	37 (50.00)		28 (51.85)		46 (45.54)	
HIV and other healthcare indicators							
Current health insurance							
No	94 (67.63)	40 (54.05)	^^^<0.001***	29 (53.70)	0.005**	62 (61.39)	^^^0.014*
Yes	45 (32.37)	34 (45.95)		25 (46.30)		39 (38.61)	
Ever taken hormones for gender affirmation							
No	49 (35.25)	24 (32.43)	0.458	13 (24.07)	0.028*	31 (30.69)	0.076
Yes	90 (64.75)	50 (67.57)		41 (75.93)		70 (69.31)	
Ever had surgery for gender affirmation							
No	112 (80.58)	58 (78.38)	0.485	42 (77.78)	0.506	79 (78.22)	0.338
Yes	27 (19.42)	16 (21.62)		12 (22.22)		22 (21.78)	
Healthcare discrimination due to transgender identity							
No	77 (55.40)	37 (50.00)	0.172	21 (38.89)	0.003**	55 (54.46)	0.716
Yes	62 (44.60)	37 (50.00)		33 (61.11)		46 (45.54)	
Ever had HIV test							
No	45 (32.37)	5 (6.76)	<0.001***	9 (16.67)	0.002**	26 (25.74)	0.006**
Yes	94 (67.63)	69 (93.24)		45 (83.33)		75 (74.26)	
Discussed HIV services with a healthcare provider							
No	41 (29.50)	4 (5.41)	<0.001***	9 (16.67)	^^^0.013*	20 (19.80)	<0.001***
Yes	98 (70.50)	70 (94.59)		45 (83.33)		81 (80.20)	
Discussed HIV services with sexual partner							
No	28 (20.14)	11 (14.86)	^^^0.137	6 (11.11)	^^^0.034*	14 (13.86)	0.003**
Yes	111 (79.86)	63 (85.14)		48 (88.89)		87 (86.14)	
HIV knowledge							
Low	69 (49.64)	68 (67.33)	0.541	36 (66.67)	0.626	68 (67.33)	0.470
High	70 (50.36)	33 (32.67)		18 (33.33)		33 (32.67)	
Avoided HIV services due to cost of services							
No	69 (49.64)	49 (48.51)	0.665	27 (50.00)	0.946	49 (48.51)	0.665
Yes	70 (50.36)	52 (51.49)		27 (50.00)		52 (51.49)	
Avoided HIV services due to distance of travel to/from healthcare facility							
No	73 (52.52)	55 (54.46)	0.456	28 (51.85)	0.900	55 (54.46)	0.456
Yes	66 (47.48)	46 (45.54)		26 (48.15)		46 (45.54)	
Avoided HIV services due to transgender identity							
No	76 (54.68)	59 (58.42)	0.149	31 (57.41)	0.606	59 (58.42)	0.149
Yes	63 (45.32)	42 (41.58)		23 (42.59)		42 (41.58)	
Avoided HIV services due to lack of LGBT anti‐discrimination policy							
No	73 (52.52)	57 (56.44)	0.132	29 (53.70)	0.823	57 (56.44)	0.132
Yes	66 (47.48)	44 (43.56)		25 (46.30)		44 (43.56)	

* *p* < 0.05.

** *p* < 0.01.

*** *p* < 0.001.

^^^ Fisher's exact test.

### 3.2 PrEP outcomes

As shown in Figure S1, 53% of the sample were aware of PrEP, 39% had discussed PrEP with their trans friends. For LAI‐PrEP, a total of 73% of the responses were very interested. A total of 23.74% of the participants were somewhat interested and 3.6% were not at all interested in LAI‐PrEP.

### 3.3 Multivariable logistic regression analyses of PrEP outcomes

In the final model (Table 2) of PrEP awareness (Model 1), higher odds of PrEP awareness were associated with respondents who were non‐Catholic (adjusted odds ratio [aOR] = 1.26, 95% confidence interval [CI] = 1.04–1.52), had an HIV test (aOR = 1.24, 95% CI = 1.03–1.49), had discussed HIV services with a healthcare provider (aOR = 1.39, 95% CI = 1.16–1.68) and had high HIV knowledge (aOR = 1.20, 95% CI = 1.02–1.41).

**Table 2:** Results of adjusted multivariable logistic regression with lasso variable selection: lasso‐selected factors associated with PrEP awareness, PrEP discussion among trans friends and interest in LAI‐PrEP

PrEP outcomes	Lasso‐selected factors	Adjusted OR (95% CI)	*p*‐Value
Model 1: PrEP awareness			
	Age		
	18–24	ref	
	25–29	1.04 (0.88–1.22)	0.589
	30–34	1.19 (0.96–1.48)	0.097
	35 years or more	1.11 (0.89–1.37)	0.331
	Past year income (Philippine Pesos)		
	No income/less than ₱10,000	ref	
	₱10,000–P20,000	1.00 (0.82–1.22)	0.970
	₱20,000–P30,000	1.14 (0.86–1.50)	0.349
	₱30,000+	1.10 (0.90–1.34)	0.325
	Religious affiliation		
	Catholic	ref	
	Non‐Catholic (e.g. Protestant, Christian)	1.26 (1.04–1.52)	0.017*
	Non‐religious	0.99 (0.74–1.32)	0.952
	Ever homelessness		
	No	ref	
	Yes	1.13 (0.97–1.32)	0.096
	Currently unemployed		
	No	ref	
	Yes	1.02 (0.86–1.21)	0.747
	General social participation		
	Low	ref	
	High	1.07 (0.93–1.22)	0.312
	Current health insurance		
	No	ref	
	Yes	1.04 (0.89–1.22)	0.566
	Healthcare discrimination due to transgender identity		
	No	ref	
	Yes	1.04 (0.89–1.22)	0.080
	Ever had HIV test		
	No	ref	
	Yes	1.24 (1.03–1.49)	0.023*
	Discussed HIV services with a healthcare provider		
	No	ref	
	Yes	1.39 (1.16–1.68)	<0.001***
	HIV knowledge		
	Low	ref	
	High	1.20 (1.02–1.41)	0.021*
Model 2: PrEP discussion among trans friends			
	Age		
	18–24	ref	
	25–29	1.07 (0.91–1.26)	0.366
	30–34	1.24 (1.01–1.55)	0.040*
	35 years or more	1.10 (0.88–1.37)	0.395
	Sexual orientation		
	Gay/lesbian	ref	
	Bisexual	1.01 (0.80–1.26)	0.921
	Straight	1.11 (0.91–1.35)	0.283
	Another sexual orientation	0.97 (0.78–1.20)	0.793
	Recent (<4 months) sex work engagement		
	No	ref	
	Yes	0.94 (0.81–1.10)	0.491
	LGBT‐specific social participation		
	Low	ref	
	High	1.02 (0.86–1.21)	0.777
	Healthcare accessibility		
	Poor/fair	ref	
	Good/excellent	0.99 (0.85–1.15)	0.911
	Current health insurance		
	No	ref	
	Yes	1.06 (0.91–1.25)	0.402
	Ever taken hormones for gender affirmation		
	No	ref	
	Yes	0.99 (0.84–1.18)	0.976
	Healthcare discrimination due to transgender identity		
	No	ref	
	Yes	1.15 (1.1–1.33)	0.044*
	Ever had HIV test		
	No	ref	
	Yes	1.40 (1.14–1.70)	0.001**
	Discussed HIV services with a healthcare provider		
	No	ref	
	Yes	1.49 (1.21–1.84)	<0.001***
	Discussed HIV services with sexual partner		
	No	ref	
	Yes	0.89 (0.74–1.08)	0.262
Model 3: Very interested in long‐acting injectable PrEP			
	Current living location		
	Metropolitan Manila	ref	
	Central Visayas	1.19 (1.01–1.41)	0.045*
	Discussed HIV services with a healthcare provider		
	No	ref	
	Yes	1.33 (1.13–1.56)	0.001**
	Discussed HIV services with sexual partner		
	No	ref	
	Yes	1.17 (1.08–1.69)	0.008**

Note: Each model/outcome ran under a non‐parametric bootstrapping procedure with 100 iterations.

Abbreviations: 95% CI, 95% confidence interval; OR, odds ratio.

* *p* < 0.05.

** *p* < 0.01.

*** *p* < 0.001.

Moreover, higher odds of PrEP discussion among trans friends (Model 2) were associated with respondents who were between 30 and 34 years older (compared to 18–24 years old) (aOR = 1.24, 95% CI = 1.01–1.55), had experienced healthcare discrimination due to transgender identity (aOR = 1.15, 95% CI = 1.1–1.33), had an HIV test (aOR = 1.40, 95% CI = 1.14–1.70) and had discussed HIV services with a healthcare provider (aOR = 1.49, 95% CI = 1.21–1.84).

Finally, the odds of being very interested in LAI‐PrEP (Model 3) were significantly higher among respondents who were currently living in Central Visayas (aOR = 1.19, 95% CI = 1.01–1.41), had discussed HIV services with a healthcare provider (aOR = 1.33, 95% CI = 1.13–1.56) and had discussed HIV services with a sexual partner (aOR = 1.17, 95% CI = 1.08–1.69).

## 4 DISCUSSION

Our study is a compelling national context for understanding the implementation factors to optimize LAI‐PrEP, based on the concentrated growth in HIV diagnoses among transfeminine people in the Philippines and the country's stated commitment to providing PrEP to priority populations.

Notably, all three PrEP outcomes were associated with discussing HIV services with a healthcare provider, reflecting a critical point for future implementation of provider‐based intervention. Although the Philippines’ Health Department has articulated a commitment to providing access to PrEP mediations for key populations in the Philippines, efforts are needed to train providers in delivering inclusive, non‐discriminatory care, including PrEP, for trans populations.

Overall, just over half of the sample had heard of PrEP. This low level of awareness might reflect the very recent approval of PrEP as part of the Philippines government's medical formulary, with a potential for higher awareness over time. Of note, those who identified as being Catholic also had lower PrEP awareness, suggesting a need to explore the possible roles of stigma and religion as contributors to HIV prevention and risk in this predominantly Catholic setting.

Even fewer participants (39%) had ever discussed PrEP with their friends. Social communication about PrEP has proven to be a challenge in many settings due to PrEP‐related stigma in many parts of the world [7]. Our findings indicated that prior experience of gender‐based discrimination in a healthcare setting was negatively associated with PrEP discussion among trans friends. This suggests a need for interventions and support systems that counteract internalized stigma and shame, for example, based on negative health‐seeking experiences, and that empower transfeminine people with comfort in openly discussing PrEP as an option for HIV prevention with their peers, particularly youths.

The majority of the sample reported being very interested in LAI‐PrEP thereby indicating a target for future intervention development around this modality. Notably, being very interested in LAI‐PrEP was more strongly endorsed by those who had previously discussed HIV services with a sexual partner, suggesting an opportunity for couples‐involved strategies to promote this modality as a strategy to promote sexual safety and satisfaction within relationships. Moreover, similar to our previous findings in daily oral PrEP among Filipina transfeminine adults [14], the current study findings also highlight the role of providers in creating gender‐affirming PrEP systems. Specifically, interactions within healthcare clinics can influence PrEP outcomes such that patients who experience negative interactions (e.g. discrimination) are more likely to report lower PrEP awareness, whereas those who are able to discuss HIV services are more likely to be interested in LAI‐PrEP, as reflected by the findings in this study. Additionally, being very interested in LAI‐PrEP was also stronger among those based outside of Metro Manila, suggesting the geographic appeal of this long‐acting modality among those who might not have proximity to the nation's most resourced hospital and public health settings. Leveraging innovative strategies and lessons learned from the Philippines’ local community‐based organizations' responses to delivering HIV services via telehealth in light of the recent COVID‐19 pandemic could be promising to integrate into future LAI‐PrEP programming [27].

Lastly, our study only examined three PrEP outcomes, and future studies must expand the scope to other outcomes along the PrEP continua (e.g. awareness, uptake, adherence and retention) [28].

### 4.1 Limitations

This study is limited in its transferability to other settings in the Philippines mainly due to our recruitment focus and eligibility criteria. For instance, our sample may not capture the diversity of trans communities in the Philippines, particularly those who do not have access to the internet, are not living in the study's recruited areas or transmasculine and non‐binary adults who are also part of trans communities [29]. Due to the cross‐sectional nature of this study's data collection design, we are unable to ascertain temporal patterns. We also deployed the survey in English, biasing our sample to those who can comprehend this language. Moreover, due to a lack of clinical guidelines for PrEP at the time this survey was conducted, we did not restrict our sample based on PrEP eligibility indicators (e.g. sexual history, serodiscordant partnerships, HIV status), as such our estimate for PrEP interest may be lower. This study lacked to incorporate new educational materials of long‐acting PrEP that refers to other options for modalities (e.g. implant, injectable) and other important attributes like injection sites (e.g. gluteal, arm) or clinic sites (e.g. HIV clinic, primary care clinic, gender clinic), a point for future research.

## 5 CONCLUSIONS

This study provides important foundational work for understanding how LAI‐PrEP may fit into the appropriate HIV prevention infrastructure among Filipina transfeminine adults. Building upon these nascent findings, we encourage future work on these fronts, especially moving beyond just research and policy goals and ensuring that other efforts actively engage with community‐based organization and their aims and needs. In doing so, the Philippines’ HIV response can continue to build towards improvements in equity among transfeminine adults and other impact sub‐populations, an important and essential goal.

## Authors’ Contributions

AJR, MIQ, JW, AA, ANB, DO and OS were involved in the conceptualization of this paper. AJR, DO and OS designed the analysis for this paper. AJR conducted the data analysis. AJR, MIQ, ANB, JG and OS analysed the data. AJR and MIQ wrote the paper. AJR, MIQ, JW, AA, JG, ANB, OG, DDF, SCU, JN, DO and OS have read, reviewed, edited and approved the final version of this manuscript.

## Competing Interests

The authors declare that they have no competing interests.

## Funding

This study was supported by the following sponsors: National Institute on Drug Abuse (R36DA048682), the Fogarty International Center (D43TW010565), Providence/Boston Center for AIDS Research (P30AI042853) and the National Institute of Mental Health (R21TW012010). Dr. Restar is a Robert Wood Johnson Foundation Health Policy Research Scholar and a Public Policy Fellow at amFAR, the Foundation for AIDS Research. Drs. Restar, Flores and Wickersham are supported by the Research Education Institute for Diverse Scholars (REIDS), funded by the National Institute of Mental Health (R25MH087217).

## Disclaimer

This article does not represent the official views of the sponsors.

## Informed Consent

Electronic informed consent was obtained from all individual participants included in the study.

## Supporting information


**Table S1**: STROBE (“STrengthening the Reporting of OBservational studies in Epidemiology”) Statement and Checklist.

**Figure S1**: PrEP awareness, PrEP discussion among trans friends and very interested in long‐acting PrEP among Filipina transfeminine adults (*n* = 139). This bar graph figure shows that 53% of the sample were aware of PrEP, 39% had discussed PrEP with their trans friends. For LAI‐PrEP, a total of 73% of the responses were very interested. A total of 23.74% of the participants were somewhat interested and 3.6% were not at all interested in LAI‐PrEP.

## Data Availability

Given that this study contains data with potentially sensitive information, data from this study are available upon request. Contact the Brown University Human Research Protection Program Institutional Review Committee (irb@brown.edu) for data requests.
