# Supplementary material for: Predictors of PrEP awareness, PrEP discussion and interest in long‐acting injectable PrEP among Filipina transfeminine adults
Source: J Int AIDS Soc. 2023 Jun 12;26(6):e26080. doi: 10.1002/jia2.26080 (PMC10258862; doi:10.1002/jia2.26080)

**S1 Fig. Figure 1.** PrEP awareness, PrEP discussion among trans friends, and very interested in long-acting PrEP among Filipina transfeminine adults (n=139).


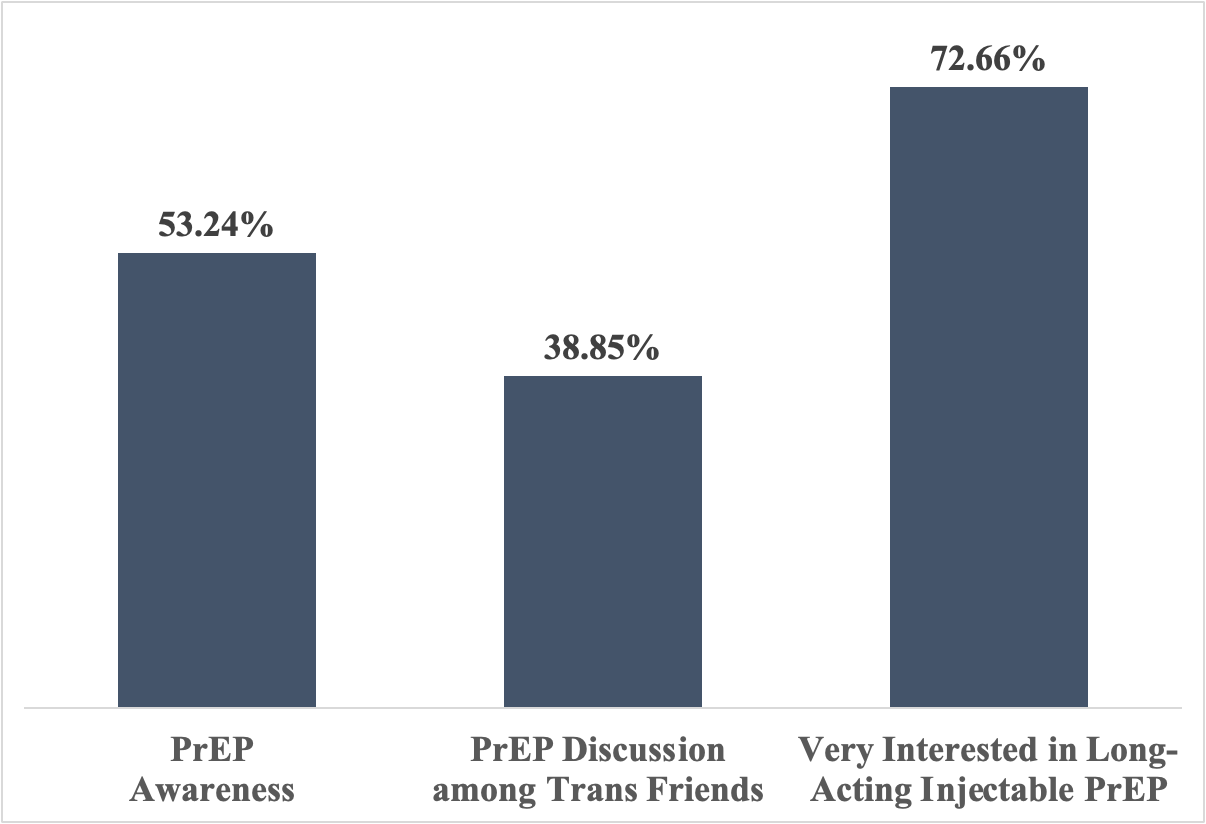

Supplement: Supplementary file 2 — Figure S1: PrEP awareness, PrEP discussion among trans friends and very interested in long‐acting PrEP among Filipina transfeminine adults (n = 139). This bar graph figure shows that 53% of the sample were aware of PrEP, 39% had discussed PrEP with their trans friends. For LAI‐PrEP, a total of 73% of the responses were very interested. A total of 23.74% of the participants were somewhat interested and 3.6% were not at all interested in LAI‐PrEP. [file JIA2-26-e26080-s001.docx]
